# A study on the impact of systematic desensitization training on competitive anxiety among Latin dance athletes

**DOI:** 10.3389/fpsyg.2024.1371501

**Published:** 2024-04-09

**Authors:** Jie Chen, Duoqi Zhou, Dan Gong, Shunli Wu, Weikai Chen

**Affiliations:** ^1^Department of Student Affairs, Jiaxing Vocational Technical College, Jiaxing, China; ^2^Sports and Art Institute, Harbin Sport University, Harbin, China; ^3^College of Physical Education, Anqing Normal University, Anqing, China; ^4^Chongqing College of International Business and Economics, College of Physical Education and Health, Chongqing, China; ^5^College of Marine Life Sciences, Ocean University of China, Qingdao, China; ^6^Department of Orthopedics, The Second Affiliated Hospital and Yuying Children’s Hosptial of Wenzhou Medical University, Wenzhou, China

**Keywords:** systematic desensitization training, psychological intervention, Latin dance training, sport, competitive anxiety

## Abstract

**Objective:**

In the domain of competitive events, Latin dance athletes have always suffered competitive anxiety, which is a prevalent and prevailing psychological facet, in pre-, intra-, and post-competitive engagements. Usually, the implementation of systematic desensitization training is an efficacious approach to reduce competitive anxiety levels in routine sports to fortify psychological resilience of athletes (like swimming, volleyball, and basketball). This study focuses on the effect of systematic desensitization training on competition anxiety in the training of Latin dancers to establish good mental ability and promote the competitive ability of athletes.

**Methodology:**

The “Sports Competition Anxiety Test Questionnaire” was used to evaluate and classify the competitive anxiety levels of 150 Latin dance athletes. Then, the top 48 participants were selected (24 in the intervention cohort and 24 in the non-intervention cohort) as the study participants after stratifying anxiety score levels from the highest to the lowest. The intervention group was treated with an 8-week psychological intervention by employing systematic desensitization training techniques (encompassing imagery desensitization and *in vivo* desensitization). The anxiety levels of the subjects were quantified by employing the “Sport Competition Trait Anxiety Inventory” (CCTAI-C) and the “Competitive State Anxiety Inventory” (CSAI-2) to scrutinize the efficacy of systematic desensitization training in regulating competitive anxiety levels among Latin dance athletes.

**Results:**

After applying systematic desensitization training, the intervention group displayed a notable reduction in sport cognitive trait anxiety. Specifically, there was a decrease of 29.37% in social evaluation anxiety, 20.31% in competition preparation anxiety, 16.98% in performance anxiety, 25.16% in failure anxiety, 34.47% in opponent’s ability anxiety, and 25.16% in injury anxiety. Moreover, for competitive state anxiety, cognitive state anxiety and somatic state anxiety decreased by 39.19 and 21.43%. The state self-confidence increased by 14.42%.

**Conclusion:**

The result indicated that systematic desensitization training not only mitigates anxiety but also positively intervenes in sports-related anxiety. Moreover, systematic desensitization training can significantly diminish competitive anxiety among Latin dance athletes to bolster confidence during competitions. Integrating desensitization training into the regular regimen of Latin dance practice has the potential to fortify dancers’ psychological resilience against anxiety.

## 1 Introduction

Anxiety is usually conceptualized as an adverse emotional state to result in distress, anger, muscular tension, and hypertension (Farber, 1948; Davies et al., 2023). Nevertheless, moderate anxiety can induce physiological responses in the body and brain, amplifying vigilance and ameliorating performance and reaction time in competitive arenas or sporting contexts (Belon, 2019). Moreover, moderate anxiety also can incite more attention of dancers in competitions, which can empower them to surmount challenges and attain peak performance (Hovenkamp-Hermelink et al., 2019).

Competitive anxiety is the stress and apprehension of athletes while they suffered worrisome occurrences before or during competitions (Butt et al., 2003). Based on Spielberger’s anxiety taxonomy, Martens classified competitive anxiety into state anxiety and trait anxiety (Martin and Hall, 1997). State anxiety denotes a transient emotional state typified by fluctuations (Endler and Kocovski, 2001). However, the trait anxiety represents a relatively enduring personality trait (Jiang et al., 2022). For athletes proficient in skill, the level of competitive anxiety will dramatically affect game outcomes (De Pero et al., 2016). During the Olympic Scientific Congress in 1984, Gruppo underscored that the psychological aspects accounted for 80% of an athlete’s success or failure in performance (Birrer and Morgan, 2010). Consequently, psychological factors and the regulation of competitive anxiety wield a pivotal role in an athlete’s performance (Binboga et al., 2012). For example, Shao et al. used imagery and systematic desensitization to adjust the competitive trait anxiety of high-level athletes at Inner Mongolia Normal University. The result showed that systematic desensitization can reduce the competition trait anxiety level of university student-athletes with serious anxiety (Guohua, 2012). Furthermore, the Venezuelan national football team has achieved remarkable results with sports psychology strategies such as systematic desensitization, relaxation, and stress coping. It can be seen that systematic desensitization training has a positive effect on competition anxiety (D’Amico and Hernández, 2017).

While competition anxiety is prevalent across various sports, its impact is particularly pronounced in skill-intensive disciplines such as Latin dance, which require a combination of physical prowess and esthetic performance (Chen, 2011). Latin dancers not only confront physical skill challenges during competition but must also navigate the effects of nervousness (Shaffer et al., 2015). The level of competition anxiety directly correlates with athletes’ performance outcomes, a phenomenon observed in Latin dance competitions as well. Thus, comprehension and regulation of competition anxiety are imperative for Latin dancers, representing a pivotal determinant of victory or defeat (Adilogullari, 2014).

Up to now, the academic field extensively deployed cognitive and behavioral intervention techniques to regulate athletes’ psychological states (Birrer and Morgan, 2010). A prominent cognitive approach includes imagery and suggestion training (Xuan, 2020; Kulshrestha et al., 2021). The behavioral strategies encompass progressive relaxation, biofeedback, and systematic desensitization methodologies (Huang et al., 2019). Systematic desensitization (Rachman, 1967), also acknowledged as systematic desensitization therapy or reciprocal inhibition (Rabinovich, 2016), represents a behavioral intervention method that gradually mitigates neurotic anxiety patterns. The hinting language and physical and psychological relaxation are used to fight against the anxiety of each level step by step under the guidance of the intervenor to achieve the purpose of relieving and eliminating anxiety. Desensitization training includes two ways of imaginary desensitization and realistic desensitization (Xiaoling et al., 2008). The basic procedure consists of three parts: muscle relaxation training, establishing fear event hierarchy, and implementation of systematic desensitization (O'Neil and Howell, 1969; Morrow et al., 1992; McGlynn et al., 2004).

This study integrates systematic desensitization training into the training regimen of Latin dance participants to investigate the effectiveness in managing competitive anxiety by randomized controlled trial. The 48 highly anxious Latin dance participants underwent psychological intervention via systematic desensitization training to employ a sports-related anxiety questionnaire. The result indicated that systematic desensitization training can effectively reduce competition state anxiety and trait anxiety of Latin dancers. The main objective of this study was to determine whether systematic desensitization training is effective in reducing competition state anxiety and trait anxiety in Latin dancers. This study bridges the gap in the application of systematic desensitization training within the realm of Latin dance, providing valuable psychological intervention insights to alleviate competitive anxiety among Latin dancers across diverse age groups.

## 2 Methods

### 2.1 Experimental subjects

**Figure 1:**
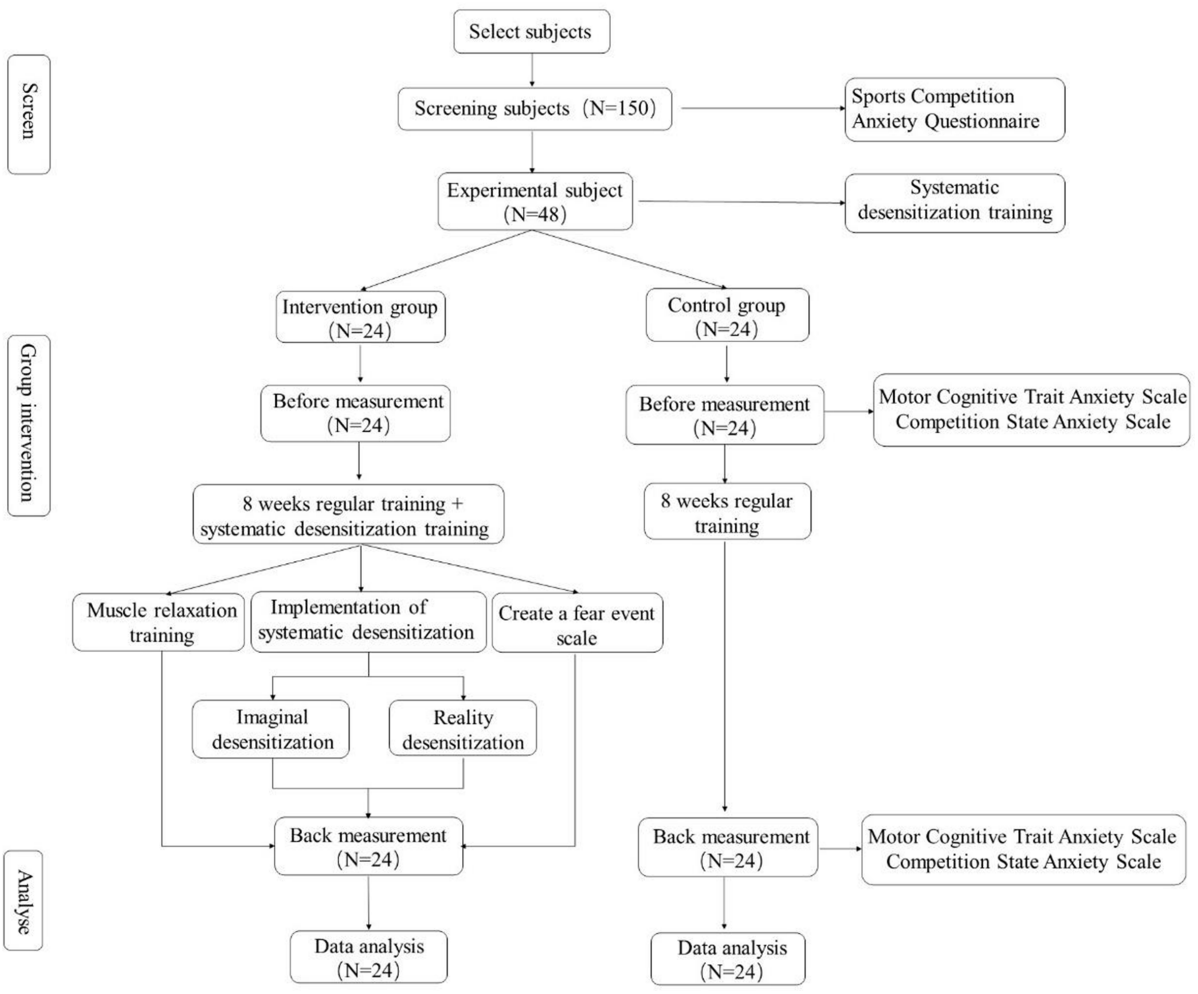
Study flow of participants.

The “Sport Competition Anxiety Test Questionnaire” (Appendix A) was employed to evaluate the competition anxiety among 150 participants (75 men and 75 women). Following the sorting based on anxiety scores, 48 Latin dance participants were selected for the research. All the participants were aged between 23 and 25 years. Among them, 24 men and 24 women were evenly distributed in the intervention group and the non-intervention group (12 men and 12 women in each group). Preceding the intervention, the subjects were classified into two cohorts: recipients of systematic desensitization training (intervention group of 24) and individuals lacking such training (non-intervention group of 24). An 8-week desensitization program, involving 16 sessions over the period (twice a week), was implemented in this study. All the subjects were recruited from the Latin Dance Elective Class after rigorous review and approval by the Ethics Committee of the Harbin Institute of Physical Education. During the recruitment process, we publicized the program through classroom announcements, posters, and emails. This study is a randomized controlled trial. All the intervention data were meticulously documented. The study visit procedure is demonstrated in Figure 1.

### 2.2 Measurement tools

Dr. Ye developed the CCTAI-C (Appendix B) to establish standardized Chinese values derived from the original scale (Ping et al., 2000). It encompasses 6 dimensions and 33 analytical indicators, individually: social evaluative anxiety, competition preparation anxiety, competitive performance anxiety, fear of failure, anxiety regarding opponents’ abilities, and injury-related anxiety. The CSAI-2 (Appendix C) was revised by Beili (1994), which was transformed by the anxiety theory by American sports psychologist Spielberger (Beili, 1994). The CSAI-2 consists of three dimensions to assess anxiety: individually, cognitive state anxiety, somatic state anxiety, and state self-confidence (with a comprehensive total of 27 assessment indicators).

### 2.3 Intervention process

#### 2.3.1 Establishment of the “fear event level scale”

Before applying psychological intervention, the tester interviews the athletes, ranking the athletes’ fears from lowest to highest (Akeb-Urai et al., 2020). They are assessed by the Fear Event Level Scale to arrange the triggers in ascending order from mild to severe. Interventions were executed based on the anxiety-provoking incidents to identify in the scale. Then, every anxiety-inducing circumstance was systematically addressed until reaching the athletes’ peak anxiety level to alleviate their competition anxiety (Lazarus and Rachman, 1957; Table 1).

**Table 1:** Fear event scale.

Serial number	Event	Level
1	Think of a lot of spectators and contestants	10
2	Think of the atmosphere at the stadium	20
3	Think of the strength of the opponent	30
4	Thinking about how unprepared you might be	40
5	Thinking that the referee may be biased	50
6	Thinking about the possibility of unexpected results	60
7	Thinking about the possibility of injury in the game	70
8	The thought of not getting good grades facing partners and coaches	80

#### 2.3.2 Muscle relaxation exercises combined with imagery systematic desensitization

The second phase of systematic desensitization is muscle relaxation training. Initially, the participants recline on a mat to foster a serene environment. The athlete is then asked to imagine the situation on the anxiety stimulus scale (Ujihara et al., 1987). Mental relaxation and progressive relaxation training combined with suggestive language were used to relax the muscles until the subjects were free of anxiety and fear of the imaginary situation and then moved to the next level (Liang et al., 2021).

#### 2.3.3 Mental relaxation method mental relaxation method

Step 1: The athlete needs to adopt a proper preparation position, such as sitting or standing, feet shoulder width apart, arms naturally down, and legs naturally upright.

Step 2: The athlete closes his eyes and mentally imagines that he is riding a “roller coaster at a constant speed.” Athletes imagine themselves starting at the beginning of the “roller coaster” and sliding along the lower end to the upper end. At this time, the athlete inhales slowly, silently saying “relax” in his heart. While the athlete appears to be at the top, he holds his breath for 3–5 s. Subsequently, the athlete begins to slide down on his own with the exhalation to reach the bottom end of the “roller coaster.” The athlete pauses and relaxes when he reaches the bottom end.

Step 3: The athlete repeats the above imaginary exercise 3 times until the tension disappears or reduces in the body. Moreover, the athlete needs to be consciously focused so that he or she can remain relaxed during practice.

#### 2.3.4 Progressive relaxation training

Step 1: The athlete needs in a supine preparatory position (lying flat on the bed, legs naturally straight, arms naturally placed in front of the body, palming facing down, and eyes gently closed).

Step 2: The athlete first makes a fist with his right hand and feels the tension for 5–8 s and then relaxes for 6–10 s to experience the relaxation. Then, the athlete makes a fist with his left hand for 5–8 s and then relaxes for 6–10 s. The above movements were repeated for 3–5 times.

Step 3: The athlete flexes the right forearm, contracting the biceps, and relaxing the body. Then, the athlete flexes his left forearm, contracting the biceps brachii, and relaxing the body. The above movements were repeated for 3–5 times.

Step 4: First, the athlete gritted his teeth and kept the tension for 5–8 s and then exhaled for 6–10 s to relax the body. Second, the athlete shrugs his shoulders, contracts his shoulder muscles to maintain tension for 5–8 s, and then exhales for 6–10 s to relax his body. Finally, the athlete takes a deep breath and holds the breath for 5–8 s and then exhales slowly to relax his body to experience the feeling of relaxation. The above movements were repeated for 3–5 times.

Step 5: The athlete contracts the abdominal muscles to maintain tension for 5–8 s and then exhales for 6–10 s to relax the body. The athlete tenses his toes for 5–8 s and then exhales for 6–10 s to relax his body. The athlete extends the ankle and holds the tension for 5–8 s and then exhales for 6–10 s to relax his body. The athlete uses abdominal breathing to hold tension for 5–8 s to bulge the abdomen, followed by exhalation for 6–10 s to relax the body and groove the abdomen. The above movements were repeated for 3–5 times.

#### 2.3.5 Relaxation training with added suggestions

After the physical relaxation exercises, the athlete begins to have an imaginary desensitization exercise with suggestive words (20–30 min each). First, the athlete adjusts himself to a comfortable sitting position, keeping the body upright. At the same time, the athlete slowly closes his eyes and takes 3–5 deep breaths. Subsequently, the athlete inhales softly to the accompaniment of light music, feeling the cool air enter the body along the nasal passages. The athlete then exhales slowly, feeling like spitting out all the tension, restlessness, and anxiety.

Step 1: The athlete imagines the situation in the first and second levels of anxiety. When the athlete feels nervous and uncomfortable, the experimenter gives the athlete a corresponding suggestion. At the same time, the athlete engages in autosuggestion (“Now focus your attention on your head, feel your scalp, forehead, and temples, be aware of your eyes, cheeks, ears, and chin. I feel very relaxed in my whole head and face. Give this relaxation to your neck, to your shoulders, to feel these areas,” “Breathe calmly and slowly, breathe very slowly and deeply,” “I feel quiet,” “I feel relaxed” and other suggestive words to intervene).

Step 2: The athlete imagines the situation of the third- and fourth-level anxiety events. The instructor gives the athlete a cue. (“Shift your attention to the arms, to your upper arms, elbows and forearms and wrists, palms and fingers, the entire shoulders and arms are completely noticed by you.” “My whole body is relaxed.” “I feel peaceful, comfortable and relaxed all over.” “I feel a kind of inner peace”).

Step 3: The athlete imagines the situation of the anxiety event in grades 5 and 6. The instructor gives the athlete a corresponding suggestion. (“Now focus your attention on your chest, your back expands as you inhale and contracts as you inhale.” “Pay attention to your abdomen, which expands as you inhale and contracts as you exhale.” “Put your attention on the spine, relax the muscles around the spine completely, release all the tension completely, and experience this feeling of relaxation,” “My mind is quiet, I do not feel anything around me.” “My arms are heavy and warm”).

Step 4: The athlete thinks of the situation of the anxiety event in grades 7 and 8. The instructor gives the athlete a cue. (“Focus on the pelvis and hips, thighs, knees, and calves.” “Bring your breath into these places and relax slowly, slowly relax.” “Continue to bring your breath to your feet and toes, the whole leg and both feet are very relaxed.” “Then focus on the whole body and imagine yourself as a scanner, from head to toe, everywhere you notice.” “Your head, torso and limbs are completely relaxed.” “The light warmth flows into my hands, my hands are warm and heavy.” “My abdomen, the middle part of my body, felt heavy and relaxed”).

### 2.4 Reality-based systematic desensitization

After the desensitization training of the imaginary system, the realistic desensitization training is carried out in the way of simulated competition. This comprehensive approach will integrate diverse influential factors encompassing audience dynamics, venue specifics, the presence of referees, and the diverse dynamics involving opponents. The experimental group felt the anxiety situation caused by the competition before the match by simulating the competition field.

Simulated Competitions Organization: Scheduled at a biweekly interval, simulated competitions will be meticulously arranged. Each preparatory phase will involve the meticulous recreation of an authentic competitive environment. This will include the utilization of a standard 12 m*12 m Latin dance competition floor, completing with requisite sound and lighting infrastructure. An approximate audience of 200 individuals will be invited to each simulated contest, aiming to replicate the ambiance characteristic of a genuine audience atmosphere. The presence of professional referees will ensure on-site evaluations, thereby safeguarding fairness and impartiality. Attire conformity to the standards of official competitions will be mandatory for all participants, because of both intervention and control group members, alongside additional participants, will be randomly assigned competitive roles for the first time. This progress will be devised for each simulated competition, including tailoring schedules, encompassing award allocations, to strive to emulate the ambiance characteristic of official competitive engagements.

Monitoring Competitors’ Anxiety Levels: A comprehensive examination of competitors’ competitive anxiety levels will be conducted both before and after the simulated competitions. Upon the conclusion of each simulated event, participants within the intervention group will receive ongoing imaginative desensitization interventions until their competitive anxiety is a successful resolution.

### 2.5 Statistical analysis

All data were measured as mean ± standard deviations. Statistical analysis was conducted by the one-way ANOVA test, considering the values with *p* ≤ 0.05 as indicating a significant difference. GraphPad Prism 8 software was used for data analysis.

## 3 Result

### 3.1 Sports cognitive trait anxiety intervention

**Figure 2:**
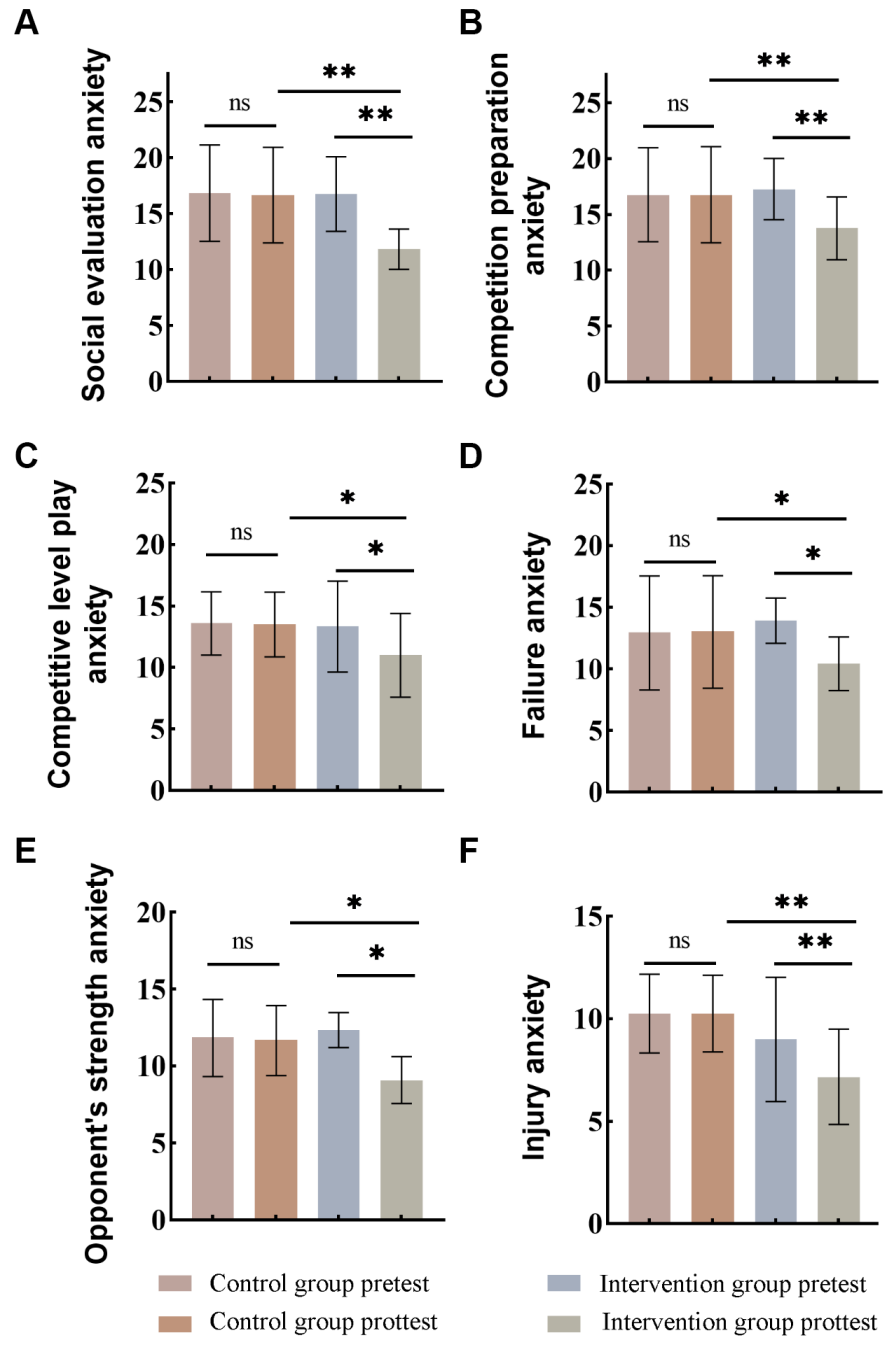
After the intervention of systematic desensitization training, the analysis of the cognitive trait anxiety in the intervention group is as follows: **(A)** Social evaluation anxiety. **(B)** Competition preparation anxiety. **(C)** Competitive level play anxiety. **(D)** Failure anxiety. **(E)** Opponent’s strength anxiety. **(F)** Injury anxiety. *p < 0.05, **p < 0.01, ***p < 0.001.

Compared with conventional training approaches, this study implemented systematic desensitization training within athletes’ schedules to alleviate the impact of competitive anxiety associated with cognitive traits in sports. Figure 2A demonstrates a comparison of social evaluative anxiety data before and after the intervention. Initial results unveiled that the pre-test social evaluative anxiety score within the intervention group was 16.75 ± 0.96. After systematic desensitization training, the intervention group experienced a substantial reduction in anxiety levels, with the post-test score notably decreasing to 11.83 ± 0.51, indicating a significant 29.37% decline. Conversely, the control group maintained consistent pre- and post-test social evaluative anxiety values of 16.83 ± 1.24 and 16.66 ± 1.29, respectively, without statistically significant variance. Figure 2B illustrates a comparative assessment of anxiety levels associated with competition preparation before and after the intervention experiment. The intervention group exhibited a decline from 17.25 ± 0.78 to 13.75 ± 0.80, representing a reduction of 20.31% in anxiety levels. Similarly, anxiety related to on-field performance decreased from 13.25 ± 1.10 to 11.00 ± 0.98 post-intervention, indicating a reduction of 16.98% (Figure 2C). Furthermore, post-intervention failure anxiety reduced from 13.91 ± 0.52 to 10.41 ± 0.62, illustrating a decrease of 25.16% in anxiety levels (Figure 2D). Anxiety associated with the opponent’s capabilities decreased from 12.33 ± 0.33 to 8.08 ± 0.28 post-intervention, demonstrating a significant reduction of 34.47% (Figure 2E). Furthermore, the anxiety related to injuries post-intervention decreased from 9.00 ± 0.87 to 7.16 ± 0.67, signifying a notable 31.56% decrease in anxiety levels (Figure 2F). Additionally, in terms of sports cognitive trait state anxiety, initial values in the intervention group spanned from 106 to 62, with an average of 82.5. After intervention, sports cognitive trait anxiety ranged from 83 to 46, averaging 63.25. Post-assessment data showed significant statistical differences between the experimental and control groups, indicating a 23.33% average reduction in anxiety levels after post-intervention. These results indicated that systematic desensitization training has remarkable efficacy in significantly mitigating sports cognitive trait anxiety of athletes, specifically in addressing anxiety linked to opponents’ capabilities.

### 3.2 Intervention for competition state anxiety

**Figure 3:**
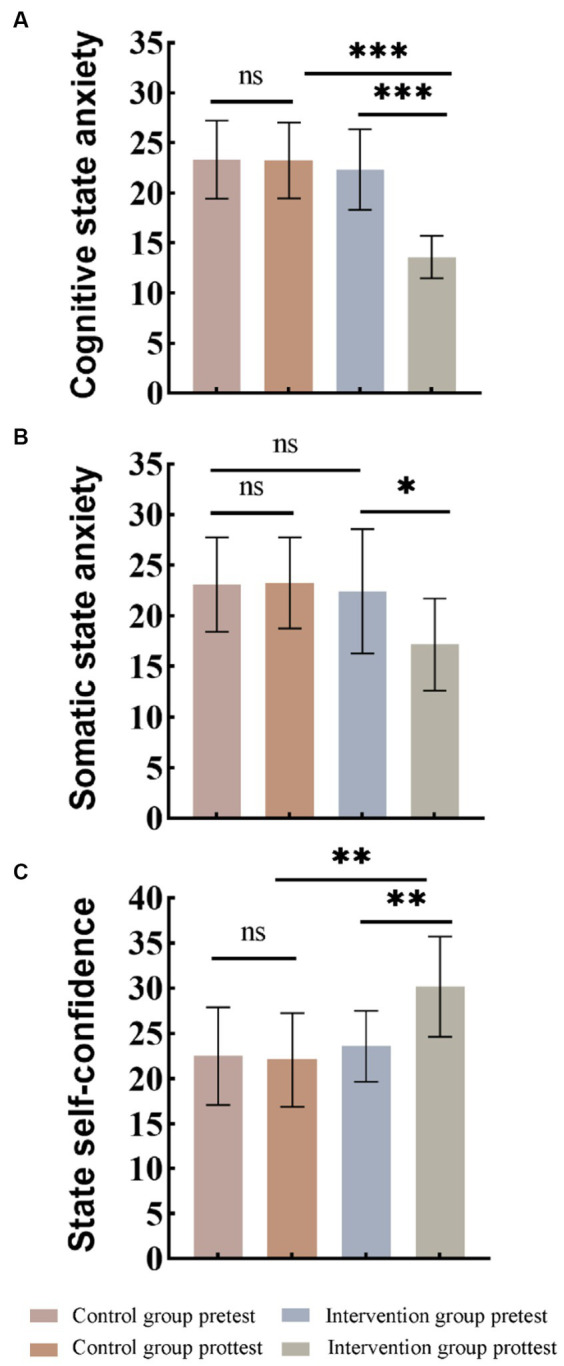
After system desensitization training intervention, the anxiety levels of the intervention group in competition were analyzed as follows: **(A)** Cognitive state anxiety. **(B)** Somatic state anxiety. **(C)** State self-confidence. *p < 0.05, **p < 0.01, ***p < 0.001.

Systematic desensitization training addresses athletes’ competitive state anxiety by regulating their cognitive state, somatic responses, and self-confidence, thereby impacting their performance in competitive settings. Figure 3A illustrates a comparative analysis of cognitive state anxiety levels before and after intervention. The results indicated that the initial cognitive state anxiety value in the intervention group was 22.33 ± 1.15. After the intervention, this value notably decreased to 13.58 ± 0.60, reflecting a remarkable reduction of 39.19% in cognitive state anxiety. In contrast, the control group showed pre-test cognitive state anxiety values of 23.33 ± 1.12 and post-test values of 23.08 ± 1.11, indicating minimal variation. Figure 3B illustrates a comparative analysis of somatic state anxiety levels before and after the intervention. The findings showed that the intervention group’s initial somatic state anxiety value was 22.41 ± 1.77, which notably decreased to 17.16 ± 1.31 after the intervention, indicating a considerable reduction of 21.43% in somatic state anxiety. Conversely, the control group exhibited pre-test values of 23.08 ± 1.34 and post-test values of 22.75 ± 1.25, indicating no statistically significant disparities. Figure 3C presents comparative analyses of state self-confidence before and after the intervention. The results suggested that the state self-confidence of intervention group initially measured 24.83 ± 1.62, rising to 28.41 ± 1.25 after the intervention, representing a notable 14.42% increase in state self-confidence. In contrast, the control group’s pre-test and post-test values were 22.50 ± 1.55 and 22.41 ± 1.57, respectively. This study emphasizes the efficacy of systematic desensitization training in alleviating cognitive state anxiety and somatic state anxiety among athletes, augmenting their self-assurance. Moreover, systematic desensitization training exhibits a more substantial impact on alleviating athletes’ cognitive state anxiety under similar conditions.

The total anxiety value in the control group decreased by 0.19% after 8 weeks, which was considered to be unchanged within the statistical error range. Conversely, the competition anxiety of athletes could be reduced by 26.17% after the systematic desensitization training intervention (Eq1). To sum up, compared to the control group, the results of the systematic desensitization training intervention reveal a notable reduction in exercise cognitive trait anxiety within the experimental group implying a favorable alteration in exercise cognitive trait anxiety attributed to systematic desensitization training.

The calculation process of the effect size of the interventions is as follows:


(1) $$\left[\left(I_{f}-I_{b}\right)/I_{f}-\left(C_{f}-C_{b}\right)/C_{f}\right]\times 100\%$$

wherein *I_f_* is the total anxiety before the intervention, *I_b_* is the total anxiety after the intervention, *C_f_* is the total anxiety before the intervention of the control group, and *C_b_* is the total anxiety of the control group after 8 weeks.

## 4 Discussion

The primary aim of this study is to amalgamate psychological intervention methodologies with Latin dance exercise, thereby investigating the impact of systematic desensitization training on competition state anxiety and exercise cognitive trait anxiety among Latin dance athletes. The physical activity intervention administered in this research endeavors not only to optimize individual physical function and form but also to regulate individual competition anxiety levels through a series of scientifically grounded training modalities and psychological counseling sessions. Concurrently, by bolstering self-efficacy and self-assurance, athletes are better equipped to confront competitions with heightened composure and confidence, thereby mitigating the adverse effects of competition anxiety. Furthermore, significant alterations in competition state anxiety (comprising cognitive state anxiety, somatic state anxiety, and state self-confidence) were observed within the experimental group post-intervention, underscoring the beneficial influence of systematic desensitization training on competition state anxiety among Latin dance athletes.

This study employs a systematic desensitization training strategy to address competition anxiety among Latin dance athletes (Tanguy et al., 2018). Compared to alternative intervention methods, systematic desensitization training can yield positive outcomes within 8 weeks. This advantage is chiefly attributed to the unique mechanism of action inherent in the intervention method. Systematic desensitization training facilitates anxiety alleviation by systematically exposing athletes to anxiety-inducing scenarios, thereby assisting them in cultivating resilience to anxiety (Chirivella and Esquiva, 2011).

In the investigation of systematic desensitization training as an intervention for competition anxiety among Latin dance athletes, several limitations persist. First, the limited participant pool of the study may impede the generalizability and reliability of the findings as they may not fully represent the diversity within the Latin dance athlete population. Secondly, while the measurement methods were underwent rigorous translation and validation processes, they predominantly relied on self-reporting, rendering them susceptible to subjective biases from participants and potentially compromising the objectivity and accuracy of the data. Future research endeavors should focus on establishing the long-term stability of the effectiveness of systematic desensitization training in reducing competition anxiety among Latin dance athletes. Initial research signals suggest the feasibility of such stability, instilling optimistic prospects for future investigations.

## 5 Conclusion

Systematic desensitization training, a psychological intervention method in psychiatry, is predominantly employed in clinical settings for psychological therapy among patients. Specifically, this method exhibits remarkable effectiveness in anxiety-related emotions. This study innovatively employs systematic desensitization training in the sports realm, investigating psychological alterations in competitive anxiety among Latin dance performers subjected to this intervention. The results suggested that the psychological intervention substantially can reduce competitive anxiety levels among Latin dance performers and significantly enhance competitive skills and achievements. Furthermore, this study also plays a significant role in guiding and reference for other sports.

## Data availability statement

The original contributions presented in the study are included in the article/Supplementary material, further inquiries can be directed to the corresponding authors.

## Ethics statement

The studies involving humans were approved by Harbin Sport University Institutional Review Board. The studies were conducted in accordance with the local legislation and institutional requirements. The participants provided their written informed consent to participate in this study.

## Author contributions

JC: Writing – review & editing, Writing – original draft, Methodology, Investigation, Formal analysis. DZ: Writing – review & editing, Formal analysis. DG: Writing – review & editing, Investigation. SW: Writing – review & editing, Visualization, Supervision, Resources, Methodology, Conceptualization. WC: Writing – review & editing, Supervision, Project administration, Methodology, Funding acquisition, Formal analysis.

## References

[ref1] Adilogullari I. (2014). The examining the effects of 12-week Latin dance exercise on social physique anxiety: the effects of 12-week Latin dance. Anthropologist 18, 421–425. doi: 10.1080/09720073.2014.11891560

[ref2] Akeb-Urai N. Kadir N. B. A. Nasir R. (2020). Mathematics anxiety and performance among college students: effectiveness of systematic desensitization treatment. Intellect. Discourse 28, 99–127.

[ref3] Beili Z. (1994). Revision of Chinese norm of state anxiety scale for sports competition. Psychol. Sci. 6, 358–362.

[ref4] Belon J. P. (2019). Anxiety and anxiety disorders. Actual. Pharm. 58, 18–22. doi: 10.1016/j.actpha.2019.09.005

[ref5] Binboga E. Guven S. Çatikkas F. Bayazit O. Tok S. (2012). Psychophysiological responses to competition and the big five personality traits. J. Hum. Kinet. 33, 187–194. doi: 10.2478/v10078-012-0057-x, PMID: 23486906 PMC3588676

[ref6] Birrer D. Morgan G. (2010). Psychological skills training is a way to enhance an athlete's performance in high-intensity sports. Scand. J. Med. Sci. Sports 20, 78–87. doi: 10.1111/j.1600-0838.2010.01188.x, PMID: 20840565

[ref7] Butt J. Weinberg R. Horn T. (2003). The intensity and directional interpretation of anxiety: fluctuations throughout competition and relationship to performance. Sport Psychol. 17, 35–54. doi: 10.1123/tsp.17.1.35

[ref8] Chen Y. Z. (2011). Influence factors and counter measures of latin players' competitive ability. Bus. Soc. 41, 415–445. doi: 10.1177/0007650302238776

[ref9] Chirivella E. C. Esquiva I. C. (2011). Psychological training in dancesport and competitive dancing. Rev. Psicol. Deporte 20, 479–490.

[ref10] D’Amico A. Hernández M. C. (2017). The Psychological Preparation of the Venezuelan National Football Team (The Burgundy Team): periods 2001–2007 and 2008–2013. Cuadernos Psicol. Deporte 17, 59–72.

[ref11] Davies M. R. Glen K. Mundy J. Ter Kuile A. R. Adey B. N. Armour C.. (2023). Factors associated with anxiety disorder comorbidity. J. Affect. Disord. 323, 280–291. doi: 10.1016/j.jad.2022.11.051, PMID: 36442657 PMC10202820

[ref12] De Pero R. Cibelli G. Cortis C. Sbriccoli P. Capranica L. Piacentini M. F. (2016). Stress related changes during TeamGym competition. J. Sports Sci. Med. 56, 639–647. PMID: 27285353

[ref13] Endler N. S. Kocovski N. L. (2001). State and trait anxiety revisited. J. Anxiety Disord. 15, 231–245. doi: 10.1016/S0887-6185(01)00060-3, PMID: 11442141

[ref14] Farber I. E. (1948). Response fixation under anxiety and non-anxiety conditions. J. Exp. Psychol. 38, 111–131. doi: 10.1037/h0055806, PMID: 18913662

[ref15] Guohua S. (2012). *Research on competitive anxiety of high-level college athletes in Inner Mongolia*.

[ref16] Hovenkamp-Hermelink J. H. M. van der Veen D. C. Voshaar R. C. O. Batelaan N. M. Penninx R. Jeronimus B. F.. (2019). Anxiety sensitivity, its stability and longitudinal association with severity of anxiety symptoms. Sci. Rep. 9:4314. doi: 10.1038/s41598-019-39931-7, PMID: 30867472 PMC6416311

[ref17] Huang X.-X. Ou P. Qian Q.-F. Huang Y. Yang S.-W. Wang Y.-X.. (2019). Clinical effect of psychological and behavioral intervention combined with biofeedback in the treatment of preschool children with attention deficit hyperactivity disorder. Zhongguo Dang Dai Er Ke Za Zhi 21, 229–233. doi: 10.7499/j.issn.1008-8830.2019.03.008 PMID: 30907345 PMC7389360

[ref18] Jiang W. Zhou X. L. Lui S. S. Y. Chan R. C. K. (2022). Emotion-regulation goals in people with high trait anxiety. PsyCh J. 11, 971–972. doi: 10.1002/pchj.608, PMID: 36184580

[ref19] Kulshrestha S. Agrawal M. Singh A. K. Ved A. (2021). Comparison of changes in cognitive functions of post-stroke patients with the computer-based cognitive intervention (PABLO system) and conventional cognitive intervention (paper-pencil method). Curr. Psychiatry Res. Rev. 17, 47–56. doi: 10.2174/2666082217666210408122228

[ref20] Lazarus A. A. Rachman S. (1957). The use of systematic desensitization in psychotherapy. S. Afr. Med. J. 31, 934–937. PMID: 13495624

[ref21] Liang D. M. Chen S. Q. Zhang W. T. Xu K. Li Y. T. Li D. H.. (2021). Investigation of a progressive relaxation training intervention on Precompetition anxiety and sports performance among collegiate student athletes. Front. Psychol. 11:541. doi: 10.3389/fpsyg.2020.617541, PMID: 33815182 PMC8009973

[ref22] Martin K. Hall C. (1997). Situational and intrapersonal moderators of sport competition state anxiety. J. Sport Behav. 20, 435–447.

[ref23] McGlynn F. D. Smitherman T. A. Gothard K. D. (2004). Comment on the status of systematic desensitization. Behav. Modif. 28, 194–205. doi: 10.1177/0145445503259414, PMID: 14997948

[ref24] Morrow G. R. Asbury R. Hammon S. Dobkin P. Caruso L. Pandya K.. (1992). Comparing the effectiveness of behavioral treatment for chemotherapy-induced nausea and vomiting when administered by oncologists, oncology nurses, and clinical psychologists. Health Psychol. 11, 250–256. doi: 10.1037/0278-6133.11.4.250, PMID: 1396493

[ref25] O'Neil D. G. Howell R. J. (1969). Three modes of hierarchy presentation in systematic desensitization therapy. Behav. Res. Ther. 7, 289–294. doi: 10.1016/0005-7967(69)90009-6, PMID: 5388061

[ref26] Ping Y. Hong H. Xiaodong X. Xiaomin S. Ni W. Ying L. (2000). A study on the standardization of sports cognitive trait anxiety scale in China. J. Chengdu Univ. Phys. Educ. 1, 33–36.

[ref27] Rabinovich M. (2016). Psychodynamic emotional regulation in view of Wolpe's desensitization model. Am. J. Psychol. 129, 65–79. doi: 10.5406/amerjpsyc.129.1.0065, PMID: 27029107

[ref28] Rachman S. (1967). Systematic desensitization. Psychol. Bull. 67, 93–103. doi: 10.1037/h0024212 6045340

[ref29] Shaffer C. T. Tenenbaum G. Eklund R. C. (2015). Implicit theories of mental skills abilities in collegiate athletes. J. Appl. Sport Psychol. 27, 464–476. doi: 10.1080/10413200.2015.1044136

[ref30] Tanguy G. Sagui E. Fabien Z. Martin-Krumm C. Canini F. Trousselard M. (2018). Anxiety and psycho-physiological stress response to competitive sport exercise. Front. Psychol. 9:1469. doi: 10.3389/fpsyg.2018.01469, PMID: 30210383 PMC6119708

[ref31] Ujihara T. Tsuga K. Akagawa Y. Tsuru H. Tatara M. (1987). A psychological approach to bruxism--application of muscle relaxation training and autogenic training. Hiroshima Daigaku Shigaku Zasshi 19, 480–485. PMID: 3333055

[ref32] Xiaoling F. U. Yan Y. Hongbo Z. Haichun S. U. N. Mingfeng Y. A. N. Lei B. I.. (2008). Effect of systematic desensitization method on reducing the surgery fear of children. Chin. J. Nurs. 43, 493–496. doi: 10.3761/j.issn.0254-1769.2008.06.003

[ref33] Xuan B. (2020). From evaluation to prediction: behavioral effects and biological markers of cognitive control intervention. Neural Plast. 2020, 1–11. doi: 10.1155/2020/1869459, PMID: 32184812 PMC7060425

