# Supplementary material for: A study on the impact of systematic desensitization training on competitive anxiety among Latin dance athletes
Source: Front Psychol. 2024 Apr 9;15:1371501. doi: 10.3389/fpsyg.2024.1371501 (PMC11037396; doi:10.3389/fpsyg.2024.1371501)
Supplement: Supplementary file 1 [file Data_Sheet_1.PDF]

Supplementary Material

Study on the Impact of Systematic Desensitization Training on Competitive Anxiety among Latin Dance Athletes

Jie Chen[1,2†], Duoqi Zhou[3†], Dan Gong[4], Shunli Wu[5]*, Weikai Chen[6]*

[1] Jiaxing Vocational Technical College, Department of Student Affairs, Jiaxing, Zhejiang, China.

[2] Harbin Sport University, Sports and Art Institute, Harbin, Heilongjiang, China.

[3] Anqing Normal University, College of Physical Education, Anqing, Anhui, China.

[4] Chongqing College of International Business and Economics, Collage of Physical Education and Health, Chongqing, China.

[5] College of Marine Life Sciences, Ocean University of China, Qingdao, Shandong, China.

[6] Department of Orthopedics, The Second Affiliated Hospital and Yuying Children's Hosptial of Wenzhou Medical University, Wenzhou, Zhejiang, China.

Appendix A

Sports Competition Anxiety Test Questionnaire

Name: Gender: Project: Project Years:

Test Date: Birth Date: Health status: Good, fair, poor

Instructions: Describe how you feel about yourself when you compete in a sport in the following 15 questions, each of which has a choice of three answers, please tick "√" in the box below. There is no right or wrong answer to the answer you choose. I hope you will not spend too much time on answering the questionnaire and will answer carefully according to your psychological self-feeling at that time.

Serial number	Questionnaire entry	Almost none	Sometimes	Often
1	I feel happy when competing with my rivals.			
2	I feel anxious before the match.			
3	I do not compete well before the match due to worries.			
4	I am a good athlete during the competition.			
5	I worry about making mistakes during the competition.			
6	I remain calm before the match.			
7	Setting goals for the competition is extremely important.			
8	I feel nauseous before the match.			
9	I notice my heart beats faster than usual before the match.			
10	I enjoy competitions that require physical energy.			
11	I feel relaxed before the match.			
12	I become nervous before the match.			
13	Team sports excite me more than individual sports.			

14	I eagerly anticipate the start of the competition.
15	I become excessively nervous before the competition.

Scoring method:

(1) Questions 1, 4, 7, 10, 13 do not count

(2) Questions 2, 3, 5, 8, 9, 12, 14, 15: A counts 1 points, B counts 2 points, C counts 3 points.

(3) Question 6,11: A counts 3 points, B counts 2 points, C counts 1 point.

The total score is calculated by multiplying the average value of the 15 questions by 10.

The higher the total score, the higher the pre-race anxiety level.

Appendix B

Sport Competition Trait Anxiety Inventory

This questionnaire lists some emotions that athletes often feel when they are facing a competition. Every athlete's mood is not exactly the same. The answers to the following questions are naturally different, so there is no "right" or "wrong" answer. Please express your personal feelings to the following questions.

There are four responses (A, B, C, D) for each question in the questionnaire, listed in the response column for you to choose one. Examples are as follows:

Question 1. I like sports. A, very suitable B, roughly suitable C, not very suitable D, very unsuitable

Which answer (A, B, C, D) is similar to your personal opinion? Select the closest answer in the answer box and mark it with a circle. For example, people who don't like sports very much circle "C".

This questionnaire lists some emotions that athletes often feel when they are facing a competition. Every athlete's mood is not exactly the same. The answers to the following questions are naturally different, so there is no "right" or "wrong" answer. Please express your personal feelings to the following questions.

There are four responses (A, B, C, D) for each question in the questionnaire, listed in the response column for you to choose one. Examples are as follows:

Question 1. I like sports. A, very suitable B, roughly suitable C, not very suitable D, very unsuitable

Which answer (A, B, C, D) is similar to your personal opinion? Select the closest answer in the answer box and mark it with a circle. For example, people who don't like sports very much circle "C".

Serial number	Questionnaire entry	Very suitable	Roughly suitable	Not very suitable	Very unsuitable
1	Concerned about the weather on the day of the competition	A	B	C	D
2	Concerned about the opponent's strengths	A	B	C	D
3	Worried about getting injured during the competition	A	B	C	D
4	Worried about performing poorly in the competition	A	B	C	D
5	Worried about not being able to perform at maximum capacity	A	B	C	D
6	Concerned about the condition of the competition venue	A	B	C	D
7	Fear of getting injured due to past injuries	A	B	C	D
8	Already thinking about losing before the competition starts	A	B	C	D
9	Concerned about disappointing the coach and teammates	A	B	C	D
10	Team cohesion is an essential element in team sports	A	B	C	D
11	Worried about not being able to perform at usual level	A	B	C	D
12	Feel uneasy when feeling equally matched with the opponent	A	B	C	D
13	More concerned about getting injured than the competition result	A	B	C	D
14	Worried about achieving good results	A	B	C	D
15	Thinking that as long as good results are achieved, injury doesn't matter	A	B	C	D
16	Worried about the consequences of losing	A	B	C	D
17	Concerned about how others perceive their competition performance	A	B	C	D
18	Feeling uneasy when competing with an opponent for the first time	A	B	C	D
19	Feeling uneasy when competing with an opponent previously defeated, thinking about the last match	A	B	C	D
20	Many outstanding athletes from various countries	A	B	C	D

	participate in Olympic competitions				
21	Worried about performing poorly in the competition	A	B	C	D
22	Concerned about how coaches and teammates perceive one's completion of tasks	A	B	C	D
23	Worried about being able to control nerves during the competition	A	B	C	D
24	Worried about insufficient training compared to usual	A	B	C	D
25	Concerned about being able to follow one's plan during the competition	A	B	C	D
26	Concerned about whether athletic condition has been adjusted	A	B	C	D
27	Worried about the audience perceiving one's competition performance as poor	A	B	C	D
28	Feeling that the competition seems to be on the verge of failure, feeling uneasy	A	B	C	D
29	Worried about unexpected occurrences during the competition	A	B	C	D
30	Victory and defeat are common in sports competitions	A	B	C	D
31	Concerned about the opponent's strength	A	B	C	D
32	Worried about whether preparatory activities have been sufficient	A	B	C	D
33	Sometimes worried about performing poorly and avoiding competition	A	B	C	D

Scoring method

The scoring method of the original score

1.A=4, B=3, C=2, D=1

2. The original scores of each measure of the Motor Cognitive Trait Anxiety Scale were calculated according to the following item numbers.

Social rating anxiety =27+17+22+21+16+28

Match preparation anxiety =26+32+25+23+24+29

Competitive level play anxiety =5+4+11+9+14

Failure anxiety =8+19+12+33+18

Strength anxiety =2+31+6+1

Injury anxiety =3+7+13+15

Polygraph score =10+20+30

Entry 15 is the reverse scoring entry, i.e. A=1, B=2, C=3, D=4. The polygraph score is used to test the truth of the answer. An answer with a polygraph score of 7 or more (=10+20+30) is considered an acceptable true answer.

Appendix C

Competitive State Anxiety Inventory

Name: Gender: Specialty: Education:

Guidance:

Here's what athletes usually say about how they feel before (or after) the game. Read each sentence carefully, then mark with a "√" the degree to which you are feeling at the moment. There are no right or wrong answers, and you don't need to spend too much time thinking about each one, but the answer is the one that best fits the situation you are feeling at the moment.

**Serial number**	**Questionnaire entry**	**Not at all**	**A bit**	**Moderate**	**Very strong**
1	I'm worried about this competition.	1	2	3	4
2	I feel nervous.	1	2	3	4
3	I am mentally stable.	1	2	3	4
4	I doubt myself.	1	2	3	4
5	I feel uneasy	1	2	3	4
6	I feel physically comfortable.	1	2	3	4
7	I'm worried that this competition won't go as well as usual.	1	2	3	4
8	I feel tense in my body.	1	2	3	4
9	I feel confident about this match.	1	2	3	4
10	I'm afraid of failing in the competition.	1	2	3	4
11	I feel tension in my stomach.	1	2	3	4
12	I am confident about this match.	1	2	3	4
13	I'm worried about not succeeding under this pressure.	1	2	3	4
14	I feel physically relaxed.	1	2	3	4
15	I am confident in facing the challenge at home.	1	2	3	4
16	I'm worried about not performing well in the competition.	1	2	3	4

17	My heart beats hard.	1	2	3	4
18	I believe I will perform excellently.	1	2	3	4
19	I'm worried about achieving my goals.	1	2	3	4
20	I feel my stomach sinking.	1	2	3	4
21	I feel mentally relaxed.	1	2	3	4
22	I'm worried that others will be disappointed in my performance.	1	2	3	4
23	My hands are cold and sweaty.	1	2	3	4
24	I am very confident because I have achieved my goals within myself.	1	2	3	4
25	I'm worried about not being able to concentrate.	1	2	3	4
26	I feel my body stiffening.	1	2	3	4
27	I am confident in completing the competition tasks under various pressures.	1	2	3	4

Scoring method: The CSAI-2 scale was scored according to 3 subscales, with a range of 9-36 points. The higher the score, the higher the cognitive state anxiety, somatic state anxiety and state confidence. The 14th question is a reverse question, which must be scored backwards, and the remaining questions are scored according to 1-4;

(1) Cognitive state anxiety: calculated by scoring questions 1, 4, 7, 10, 13, 16, 19, 22 and 25.

(2) Somatic state anxiety: calculated by the scores of questions 2, 5, 8, 11, 14, 17, 20, 23 and 26.

(3) State confidence: calculated by the scores of questions 3, 6, 9, 12, 15, 18, 21, 24 and 27.

If one question on each subscale is not answered, the questionnaire will still be scored, but if two or more questions are not answered, the questionnaire will not be valid. When a subscale is missing one question, the average score of the eight questions answered can be calculated first, and then the average score is multiplied by 9 to take its integer.
